# Transabdominal Motor Action Potential Monitoring of Pedicle Screw Placement During Minimally Invasive Spinal Procedures: A Case Study

**DOI:** 10.7759/cureus.9497

**Published:** 2020-07-31

**Authors:** Anisha Narayan, Sandy Taylor, William Taylor

**Affiliations:** 1 Department of Neurosurgery, University of California San Diego, La Jolla, USA

**Keywords:** neuromonitoring, tamap, pedicle screw, minimally invasive, emg

## Abstract

Precise pedicle screw placement is a critical skill during minimally invasive spinal surgeries but can pose various challenges. Techniques such as electromyography (EMG) have been traditionally utilized for this purpose but have several shortcomings. Transabdominal motor action potential (TaMAP) has been examined as a possible effective neuromonitoring alternative and is hypothesized to provide important data on symptomatic malpositioned pedicle screws. The current study seeks to determine whether TaMAP may be an advantageous technique in the neuromonitoring of percutaneous pedicle screw placement during minimally invasive spinal procedures. The methodology involved recording TaMAP signals at the outset and the conclusion of spinal surgical procedures in human participants, for which comparisons were made of pre- and post-operative data. Results revealed that TaMAP signals remained stable during accurate pedicle screw placement and degraded during a case of inaccurate placement, for which initial misplaced hardware altered the depolarization threshold and resulted in substantial signal alteration. These results suggest that TaMAP, which is stable, repeatable, and reflects real-time information, can potentially be used as a reliable and more precise indication of accuracy in pedicle screw placement during spinal surgeries. This is the first TaMAP study conducted in human participants.

## Introduction

Background

Precise placement of percutaneous pedicle screws is a critical technique for minimally invasive spinal procedures [1]. Although minimally invasive methods have been shown to have advantages over open surgical techniques, resulting in decreased muscle damage and blood loss, determination of the pedicle screw trajectory may be particularly challenging due to the difficulty in visualizing it, leading to nerve injuries [2]. Misplaced pedicle screws are a common, potentially detrimental medical issue, yet methods to monitor accurate placement have significant drawbacks and are currently lacking standardization [2-3]. The current study suggests a novel method to improve neuromonitoring during pedicle screw placement using the transabdominal motor action potential (TaMAP) technique. This neurophysiological tool can assess the functional integrity of individual nerve roots in real-time and may help surgeons recognize impending neural injuries that cannot be detected by traditionally used techniques such as electromyography (EMG) [4]. Back pain is one of the most common conditions worldwide, suggested to affect 80% of adults during their lifetime; thus, safely monitoring screw placement for treating back pathologies is of crucial importance [5].

Intraoperative spinal procedures utilizing pedicle screws can treat a variety of conditions causing back pain such as spinal trauma, degenerative spinal diseases, scoliosis, and spondylolisthesis [6]. In minimally invasive spinal surgeries, pedicle screw placement is the widely used method to facilitate the stabilization of the spine [7]. This technique involves the attachment of rods and plates to the screws to allow for fixation to the pedicles, particularly in the cervical and thoracic regions, which can vary anatomically in location and size. Although the use of minimally invasive percutaneous techniques confers several advantages, it can obscure landmarks causing difficulty with the positioning of the screw, ultimately requiring revision surgeries [8].

Injuries during pedicle screw placement

Attempts to accurately position pedicle screws can pose several challenges. For example, the corridor of the pedicle is narrow and can damage adjacent vital structures when breached [9]. Studies have observed that minimally invasive pedicle screw placement presents a risk of transecting the medial nerve branch or nerve roots at the level of the lumbar spine, which can lead to devastating consequences such as radiculopathies, pseudoarthrosis, and paralysis [10]. Additionally, transection can lead to denervation of the fascicles of important muscles involved in spine stabilization such as the multifidus muscle [10]. One study suggested that the risk of damage due to misplaced screws was around 18% [11] and another showed the risk to be as high as 20% [1]. Another comprehensive study examining risks in thoracic spine pedicle screw positioning showed associations with moderate cortical and medial wall perforation [3]. Additionally, research found symptoms of late-onset pain in nerves surrounding the insertion sites and associated neurological weakness [12]. Neurological risks can be increased for patients who have weaker vertebra such as those who are elderly and/or have osteoporosis [12]. According to a 2017 study by Goerres et al., nearly 500,000 spinal fusion cases are performed yearly in the US alone, thus even a low error rate can be extremely costly, translating to tens of thousands of cases with neurological complications and unnecessary repeat surgeries [13].

Drawbacks to the use of EMG alone during screw placement

Prior studies demonstrated the effectiveness of using EMG responses generated by electrical stimulation. One such study, which was conducted in the abdominal and leg muscles of sheep, showed that EMG could be effectively used to indicate the pedicle screw position [2]. EMG has been utilized as a technique to facilitate accurate pedicle screw placement for both the thoracic and lumbar spine [2,4], and its ability to detect breaches in the pedicle cortex is advantageous to imaging techniques during minimally invasive procedures [14]. However, lateral pedicle breaches are difficult to identify using EMG alone [14]. EMG use can lead to false positives and has low sensitivity, with one paper reporting 22% of misplaced screws to be undetectable by EMG methods [15]. Moreover, studies have found that while nerve dysfunction can be detected by EMG, other injuries such as vascular events cannot [16]. Thus, TaMAP has been suggested as a newer, more recently developed neuromonitoring alternative.

TaMAP as a neuromonitoring technique

TaMAP is a relatively new form of neurophysiological monitoring that has shown effectiveness in detecting inadvertent iatrogenic nerve damage due to spinal procedures [17]. This intraoperative method monitors the integrity of nerves by electrode stimulation across the abdomen at the T12 level to trigger a response within corresponding muscles, as has been demonstrated in ovine models [17]. TaMAP recordings can be obtained as a function of stimulus current, offering real-time functional feedback. Studies on porcine models have shown TaMAP to be a beneficial neuromonitoring alternative to the complications and limitations caused by a previously used technique transcranial motor evoked potential (TcMEP) [17-18]. Thus, the current study seeks to validate the use of TaMAP as a safer, more effective alternative to prior methods and is the first to examine this technique in human participants.

## Materials and methods

**Figure 1:**
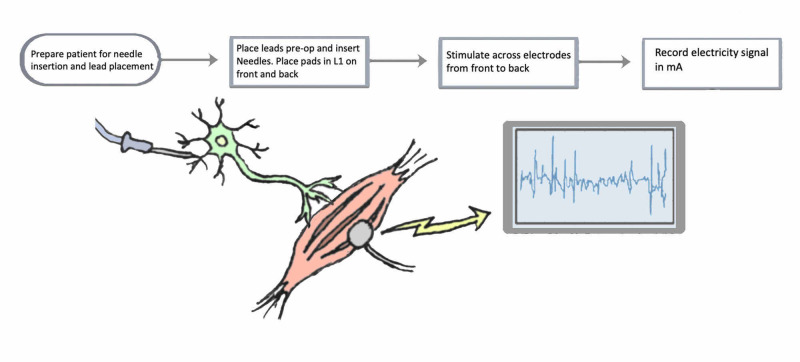
TaMAP workflow 1. Prepare patient; place leads onto specific muscle groups preoperatively and insert needles. Electrodes are connected to wires that relay to the signal recording box. 2. Two pads are placed on the patient: one on L1 anteriorly over the abdomen, and one on L1 posteriorly. 3. Stimulate across electrodes from anterior to posterior. Stimulation is picked up in the spinal cord. 4. Record distally the amount of electricity (in mA) required to depolarize muscle in each muscle group (standard action potential). Note: if the pedicle screw is malpositioned and impinges on a nerve, the nerve and muscle irritation will be reflected as abnormalities on TaMAP recording. TaMAP: transabdominal motor action potential Image credit: Anisha Narayan

Neuromonitoring was conducted during the placement of percutaneous pedicle screws. Out of the first 50 patients who underwent minimally invasive spinal procedures, only six underwent fusion procedures involving pedicle screw placement, including extreme lateral interbody fusion (XLIF), transforaminal lumbar interbody fusion (TLIF), and posterior spinal fusion (PSF). These patients were subjected to TaMAP recording in conjunction with routine EMG monitoring. All patients who underwent percutaneous pedicle screw placement prior to a minimum three-month follow-up were included in the analysis. Baseline TaMAPs were acquired prior to incision, and final responses were again examined upon wound closure. Data were obtained from preoperative and postoperative neurological exams (see Figure 1).

Responses were measured in the adductor magnus, gastrocnemius, vastus medialis, tibialis anterior, and biceps femoris muscles. TaMAP data were measured as a function of stimulus current (in mA and µVpp). Responses were measured by the level of current required to depolarize the nerve and subsequent muscle (see Tables 1-2). Latency and amplitude were also measured.

**Table 1:** TaMAP signals at the beginning of the procedure TaMAP signals recorded in mA and/or µVpp at the beginning of the procedure in the right adductor magnus, left med. gastrocnemius, right med. gastrocnemius, left vastus medialis, right vastus medialis, left tibialis anterior, right tibialis anterior, left biceps femoris, and right biceps femoris. The screw was misplaced for the right tibialis anterior, resulting in a recorded value of 1128 mA. TaMAP: transabdominal motor action potential

Subject No.	R_Ad_Mag_uV	L_Med_Gast_AMPS	L_Med_Gast_uV	R_Med_Gast_AMPS	R_Med_Gast_uV	L_Vast_Med_AMPS	L_Vast_Med_uV	R_Vast_Med_AMPS
1	500	220	400	57	650	49	400	34
2	900	48	650	67	1050	34	700	31
3	550	42	650	52	400	66	350	105
4	OFF	OFF	OFF	OFF	400	233	450	42
5	No response	No response	550	512	700	36	600	45
6	500	83	No response	No response	550	38	550	47
	R_Vast_Med_uV	L_Tib_Ant_AMPS	L_Tib_Ant_uV	R_Tib_Ant_AMPS	R_Tib_Ant_uV	L_Bic_Fem_AMPS	L_Bic_Fem_uV	R_Bic_Fem_AMPS
1	450	50	450	50	OFF	OFF	OFF	OFF
2	800	33	800	51	OFF	OFF	OFF	OFF
3	350	102	500	80	350	32	300	66
4	400	108	450	43	400	30	600	51
5	600	163	550	1128	750	35	550	109
6	300	43	550	59	550	41	500	37

**Table 2:** TaMAP signals at the end of the procedure TaMAP signals recorded in mA and/or µVpp at the end of the procedure in the right adductor magnus, left med. gastrocnemius, right med. gastrocnemius, left vastus medialis, right vastus medialis, left tibialis anterior, right tibialis anterior, left biceps femoris, and right biceps femoris. TaMAP: transabdominal motor action potential

Subject No.	R_Ad_Mag_uV	L_Med_Gast_AMPS	L_Med_Gast_uV	R_Med_Gast_AMPS	R_Med_Gast_uV	L_Vast_Med_AMPS	L_Vast_Med_uV	R_Vast_Med_AMPS
1								
2	850	48	650	81	1100	32	650	32
3	550	40	650	49	350	32	650	109
4	OFF	OFF	OFF	OFF	400	206	650	314
5	No response	No response	550	123	750	33	650	33
6	500	228	No response	No reponse	500	41	650	55
	R_Vast_Med_uV	L_Tib_Ant_AMPS	L_Tib_Ant_uV	R_Tib_Ant_AMPS	R_Tib_Ant_uV	L_Bic_Fem_AMPS	L_Bic_Fem_uV	R_Bic_Fem_AMPS
1								
2	800	38	800	38	OFF	OFF	OFF	OFF
3	400	268	500	80	350	34	300	67
4	450	115	450	32	400	291	400	57
5	550	86	550	58	750	45	650	83
6	300	46	500	43	500	46	500	30

## Results

TaMAP signals were recorded before and after placement in each muscle group (adductor magnus, gastrocnemius, vastus medialis, tibialis anterior, and biceps femoris). All patients showed detectable TaMAPs that were stable and repeatable during the procedure. Stimulation currents over 600 mAmps were observed in patients with increased body mass index (BMI) and in deeper muscle recordings. Action potential for individual muscle groups was monitored for amplitude and latency. The TaMAP depolarization value remained unchanged, with appropriate pedicle screw placement in any of the patients. In a single case, an L4 pedicle screw was misplaced medially, and an increase in depolarization in that root was identified. Notably, after the screw was replaced in the appropriate location, the stimulation value returned to normal.

## Discussion

According to results, out of the six patients who received minimally invasive spinal fusion procedures involving pedicle screw placement for the aforementioned muscle groups, only one pedicle screw was inaccurately placed, resulting in a drastic activity spike. As seen in Figure 2, the pedicle screw was misplaced for the right tibialis anterior, resulting in a recorded measurement of 1128 mA. When the screw was replaced in the correct position, the signal returned to the normal value. This is in concordance with our predictions, as misplaced pedicle screws would be expected to result in an increased value if the nerve is impinged, given that an irritated nerve requires more energy to reach depolarization [19]. Thus, our findings support the efficacy of TaMAP as an informative tool in neuromonitoring.

The use of this monitoring system is relatively safe and requires no change in anesthetic technique, as it reflects a peripheral response (as opposed to a central motor pathway), other than avoiding paralytic agents. Although EMG methods, such as triggered EMG (trEMG), involve stimulating the pedicle directly with an electric probe [20-21], the utilization of TaMAP adds functional information regarding nerve integrity. Unlike EMG alone, TaMAP provides detail that often remains undetected by other modalities and conveys whether the nerve has been compressed or injured, rather than merely the muscle.

Additional studies are needed to further corroborate these findings and continue to examine the benefits of this technique. There is currently only one other study to our knowledge discussing TaMAP, as it is a relatively novel tool [17]. Given that the current study involved only a case of a malpositioned screw, future research should involve an experimental follow-up with a larger, more comprehensive set of data points. Subsequent studies should focus on further refining the technique, validating physician protocols, and developing clinical guidelines.

## Conclusions

Neuromonitoring during the placement of pedicle screws, especially those placed percutaneously, has recently become more standard. The current study suggests a unique application for the novel transabdominal motor action potential procedures during minimally invasive spinal procedures. This technique allows the surgeon to identify nerve compression, which may be symptomatic but missed by other modalities such as EMG alone. The current study provides support for the possibility of TaMAP as a useful neuromonitoring technique in minimally invasive spinal surgeries and is the first TaMAP study conducted in human participants.
